# Automated monitoring of honey bees with barcodes and artificial intelligence reveals two distinct social networks from a single affiliative behavior

**DOI:** 10.1038/s41598-022-26825-4

**Published:** 2023-01-27

**Authors:** Tim Gernat, Tobias Jagla, Beryl M. Jones, Martin Middendorf, Gene E. Robinson

**Affiliations:** 1 Carl R. Woese Institute for Genomic Biology, University of Illinois at Urbana-Champaign, 1206 West Gregory Drive, Urbana, IL 61801 USA; 2 Swarm Intelligence and Complex Systems Group, Department of Computer Science, Leipzig University, Augustusplatz 10, 04109 Leipzig, Germany; 3 Neuroscience Program, University of Illinois at Urbana-Champaign, 505 South Goodwin Avenue, Urbana, IL 61801 USA; 4 Department of Entomology, University of Illinois at Urbana-Champaign, 320 Morrill Hall, Urbana, IL 61801 USA; 5 Present Address: Department of Ecology and Evolutionary Biology, Princeton University, 106A Guyot Lane, Princeton, NJ 08544 USA; 6 Present Address: Lewis-Sigler Institute for Integrative Genomics, Princeton University, South Drive, Princeton, NJ 08544 USA

**Keywords:** Image processing, Machine learning, Behavioural methods, Complex networks, Dynamic networks

## Abstract

Barcode-based tracking of individuals is revolutionizing animal behavior studies, but further progress hinges on whether in addition to determining an individual’s location, specific behaviors can be identified and monitored. We achieve this goal using information from the barcodes to identify tightly bounded image regions that potentially show the behavior of interest. These image regions are then analyzed with convolutional neural networks to verify that the behavior occurred. When applied to a challenging test case, detecting social liquid transfer (trophallaxis) in the honey bee hive, this approach yielded a 67% higher sensitivity and an 11% lower error rate than the best detector for honey bee trophallaxis so far. We were furthermore able to automatically detect whether a bee donates or receives liquid, which previously required manual observations. By applying our trophallaxis detector to recordings from three honey bee colonies and performing simulations, we discovered that liquid exchanges among bees generate two distinct social networks with different transmission capabilities. Finally, we demonstrate that our approach generalizes to detecting other specific behaviors. We envision that its broad application will enable automatic, high-resolution behavioral studies that address a broad range of previously intractable questions in evolutionary biology, ethology, neuroscience, and molecular biology.

## Introduction

Barcode-based tracking makes it possible to automatically distinguish hundreds of individuals in digital videos, and to record their location and heading direction over long time periods at a high spatiotemporal resolution^1–4^. This is otherwise not possible, and has already generated a wealth of individualized data that is transforming how ethologists study the behavior of animals, especially for species that naturally interact in large collectives, such as ants and honey bees^4–8^.

In addition to knowing where individuals are located, it is often necessary to also know what they are doing, to understand both individual and group-level behavior and its neural and molecular underpinnings. However, with the exception of locomotion, barcodes are unable to automatically generate behavioral information directly. When studying other behaviors, researchers therefore tend to resort to proxies that infer coarse-grained behavioral states from changes in the location and orientation of an individual’s barcode, and social interactions from the relative position of individuals to each other^2–7,9^. Such proxies have a limited capacity for distinguishing specific behaviors^10^ and may thus result in high error rates.

Convolutional neural networks (CNNs) are a promising technology for developing detectors for specific behaviors. They can be trained to accurately identify digital images that show a particular object, and learn independently which features of the object are most diagnostic^11^. CNNs have been used for tracking animals^12–14^, estimating their pose^15–18^, and detecting behaviors performed in isolation^19,20^ or in small groups^20,21^. However, they have not yet been widely employed to identify behavior in videos showing hundreds of closely interacting individuals. Obstacles to more widespread adoption of CNNs include issues that arise when detecting small objects in large scenes^22^, detecting dense or partially occluded objects^22^, and assigning detected behaviors to the correct individuals.

We present a method that combines CNNs with barcode-based tracking to accurately identify specific behaviors in large animal collectives. Complementing the innovations in ref.^23,24^, our key contribution lies in combining tracking information, such as an animal’s location and orientation, with domain knowledge about the behavior of interest to perform a precise yet computationally inexpensive region proposal that acts as an attention mechanism for the CNN. In addition, we leverage information from the barcodes to simplify the behavior classification task by rotating the proposed image regions to correspond to a predefined reference frame. Using this approach, we designed a proof-of-principle detector for mouth-to-mouth liquid transfer (trophallaxis) and then used it to gain new insights into this still poorly understood behavior. We also designed a detector for egg-laying to show that our methodology can be applied to very different behaviors.

## Results and discussion

**Figure 1:**
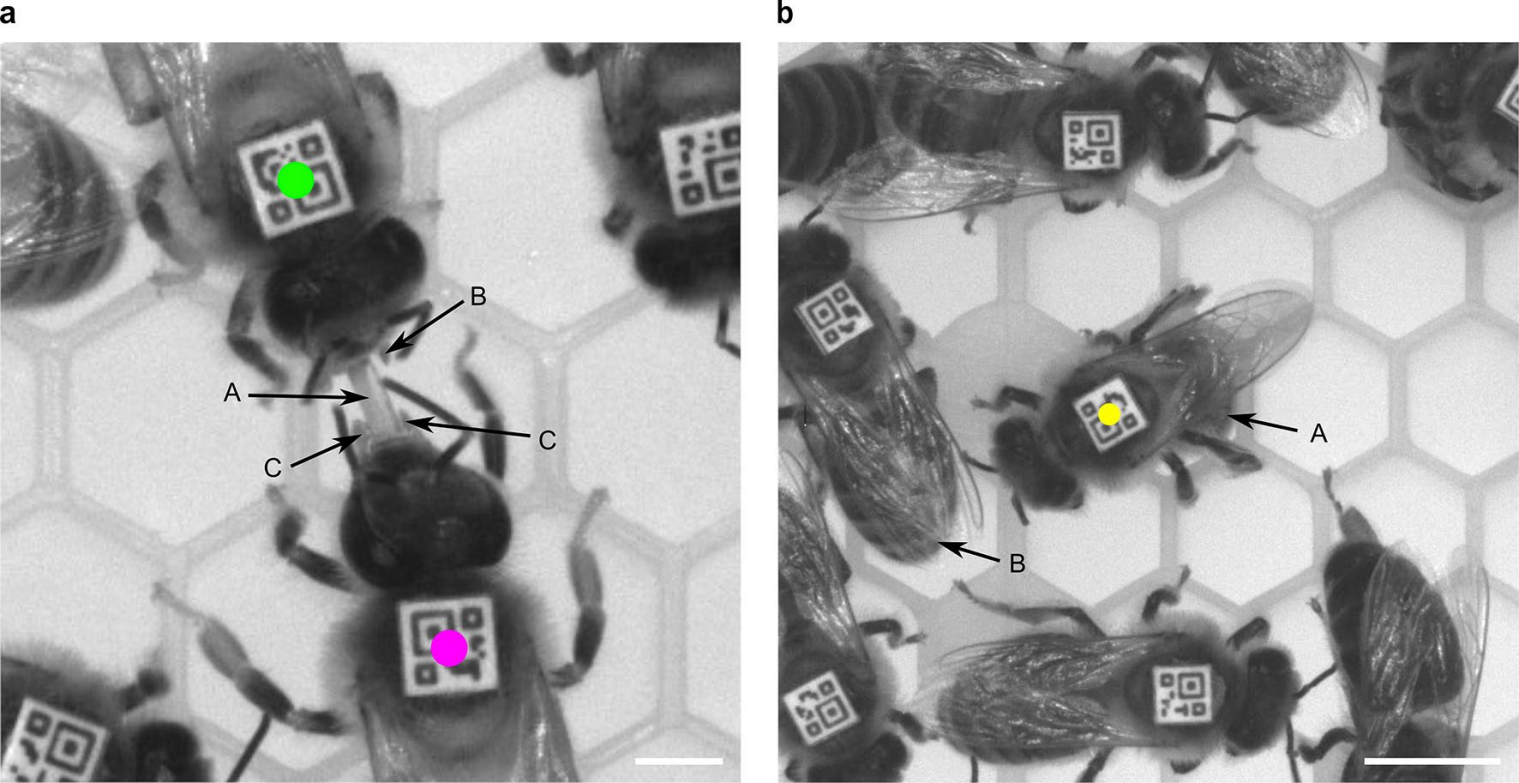
Examples of trophallaxis and egg-laying, illustrating the stark differences between these two behaviors. (**a**) In honey bees, trophallaxis is a social behavior during which two individuals orally exchange a liquid containing nutrients, hormones, and signaling molecules. One of the two bees (green) acts as the donor while the other bee (magenta) acts as the recipient. These roles are maintained for the duration of the behavior. Our automatic trophallaxis detector identifies this behavior by recognizing that the recipient has placed her proboscis (A) between the open mandibles (B) of the donor. In this image, only one of the donor’s mandibles is clearly visible. The other one is partially hidden by her right antenna. To distinguish donor and recipient, our detector may take advantage of the fact that the mandibles (C) of the recipient are closely aligned with her proboscis while those of the recipient are wide open. In addition, the head of the recipient appears to be bigger and shaped differently, because she has tilted it back to be able to extend her proboscis. Note that the proboscis and a cell wall of the plastic honeycomb look very similar; whether the mandibles are visible depends on how a bee holds her head and antennae. Scale bar, 2 mm. (**b**) Egg-laying is a solitary behavior during which an individual bee (yellow) places an egg inside a honeycomb cell. To position the egg, the bee inserts her abdomen into a cell and remains stationary while releasing the egg. Our automatic detector identifies this behavior by distinguishing bees whose abdomens are hidden (A) from those whose abdomens are visible (B). Note that under more crowded conditions than in this image it will be more challenging to make this distinction, because the space behind an egg-layer might be (partially) occupied by the abdomen of another bee. Scale bar, 5 mm.

**Figure 2:**
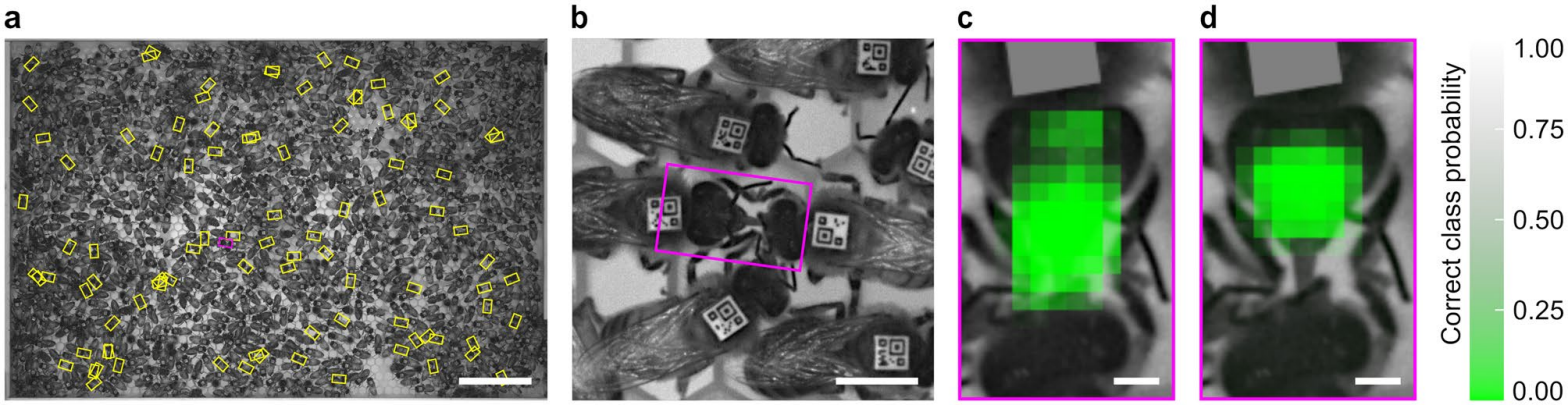
Automatic trophallaxis detection and recipient identification. (**a**) Typical image captured by the honey bee tracking rig, showing barcoded bees inside the observation hive. Rectangles outline image regions identified by our barcode-based region proposal procedure for trophallaxis. Scale bar, 5 cm. (**b**) Zoom-in on the magenta region proposal in (**a**), which shows the head, forelegs and part of the thorax of two bees that engage in trophallaxis. Scale bar, 5 mm. (**c**) CNN input image created from the region proposal in (**b**) superimposed with a map of the output of the CNN for detecting the occurrence of trophallaxis, as a function of the position of a grey square that occludes some pixels (see “Methods”). A false negative from the input image is more likely if pixels corresponding to the mouthparts and proboscis are obscured, indicating that the CNN is able to distinguish salient features of trophallaxis from the background. Scale bar, 1 mm. (**d**) The same input image as in (**c**) but superimposed with a map of the correct class probability for recipient identification. This map suggests that the CNN for identifying the recipient relies on visual cues obtained from the mouthparts of the recipient, which, unlike those of the donor, are always visible and closely aligned with the proboscis. Scale bar, 1 mm.

Trophallaxis is an important social behavior during which two adult worker honey bees touch each other with their antennae while orally transferring liquid containing food^25^ and signaling molecules^26^ (Fig. 1a). This behavior is challenging to detect automatically because honey bee colonies contain hundreds to tens of thousands of individuals densely crowded together (Fig. 2a). Moreover, owing to the small size of the honey bee, a detector needs to focus on millimeter-sized body parts, such as the mouthparts, to distinguish trophallaxis from visually similar behaviors, such as antennation.

To identify trophallaxis, our automatic detector first uses location and heading direction, obtained from each bee’s barcode, to select pairs of bees that are in the proper position relative to each other to perform the behavior (Supplementary Fig. 1). For each pair, the detector then estimates a rectangular image region that shows only the bees’ mouthparts and head (Fig. 2a,b), which will be examined to verify trophallaxis. This region proposal excludes 97.9 ± 1.0% (mean ± standard deviation, n = 300) of a video frame from further consideration, thus increasing the detector’s computational efficiency and reducing its false positive rate. Image regions are then independently preprocessed and scored with a CNN that was trained to estimate the probability of a region showing trophallaxis (Supplementary Table 1 and Fig. 2c). During post-processing, these probabilities are integrated across successive video frames to determine when trophallaxis begins and ends.

Performance measurements (Table 1) showed that our trophallaxis detector outperforms the state of the art^8,24^. Its Matthews correlation coefficient (MCC) is 0.89, 0.28 higher than that of the best automatic honey bee trophallaxis detector so far^8^. Most of this improvement is due to a 67% higher sensitivity and an 11% higher positive predictive value, which means that our detector identifies more trophallaxis interactions and generates fewer false positives. Unlike other honey bee trophallaxis detectors^8,9^, it also accurately scores interactions between adult worker bees and the queen. Moreover, at 55 megapixel/s, it processes videos 16 times faster than the reference detector^8^, and thus enables larger-scale experiments.

**Table 1:** Detailed detection and runtime performance estimates.

Detector	F_1_ score	Sensitivity	Specificity	PPV	NPV	Speed (MP/s)
Trophallaxis	0.89	0.89	1.00	0.90	1.00	55.38
Egg-laying	0.75	0.63	1.00	0.93	1.00	20.87

Note that the runtime estimate for trophallaxis detection includes detecting the occurrence of trophallaxis as well as identifying the recipient. *PPV* positive predictive value, *NPV* negative predictive value.

When studying social behavior, it is often important to know the role played by each individual, i.e., who is the “donor” and who is the “recipient” of a particular behavioral interaction. For honey bee trophallaxis, this involves distinguishing the individual that has only opened her mouthparts (donor) from the one that has also extended her proboscis (recipient) (Fig. 1). Previously described automatic trophallaxis detectors are either unable to determine the direction of liquid transfer^8,9,23^ or require fluorescence-labeled liquids and a more complex tracking system to do so^24^. To establish the liquid transfer direction directly from videos, we trained a second CNN (Supplementary Table 1 and Fig. 2d) that identifies the liquid recipient, and operated it in parallel with the CNN that detects the occurrence of trophallaxis. When applied to automatically identified trophallaxis partners, this CNN has a MCC of 0.97, which means that in almost all cases it correctly determined a bee’s trophallactic role.

**Figure 3:**
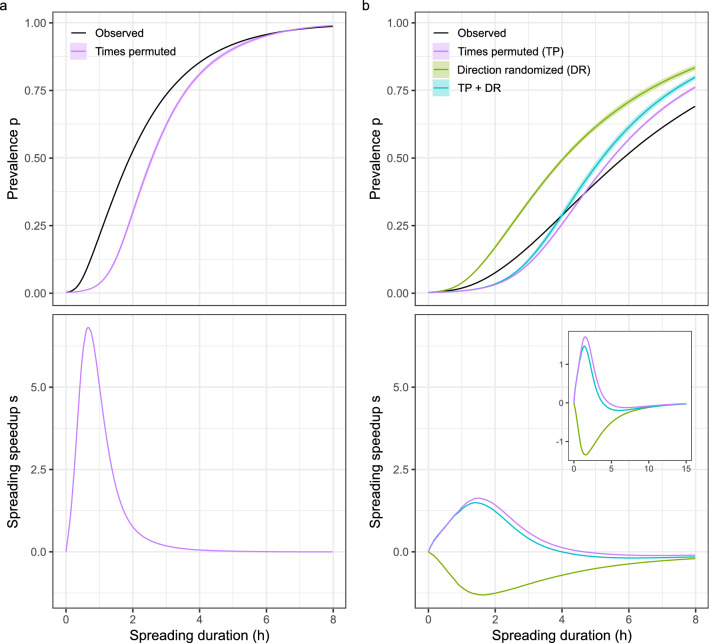
Simulated spreading though honey bee trophallaxis networks. Panels show data from Trial 1; see Supplementary Figs. 2, 3 for Trial 2 and 3, respectively, which yielded similar results. (**a**) Undirected spreading results, modeling transmission via physical contacts during trophallaxis. Top panel: Average fraction of “infected” bees (prevalence) as a function of spreading duration. Black line, prevalence in the observed trophallaxis network; magenta line, mean prevalence, averaged across 5 temporally randomized reference networks; magenta band, point-wise 95% confidence interval. Bottom panel: Spreading speedup, corresponding to the normalized difference between the prevalence in the observed network and the mean prevalence across the 5 temporally randomized reference networks, as a function of spreading duration. The spreading speedup is positive if the prevalence in the observed trophallaxis network is higher than in the randomized reference networks, zero if there is no difference, and negative if it is lower. Simulation results indicate that the temporal pattern of trophallaxis accelerates undirected spreading. (**b**) Directed spreading results, modeling liquid flow. Panels as in **a**. Green, prevalence and spreading speedup in directionally randomized reference networks; turquoise, prevalence and spreading speedup in temporally and directionally randomized reference networks. Simulation results indicate that the temporal pattern of trophallaxis accelerates directed spreading during the early phase of the spreading process, but inhibits it later. The directional nature of trophallaxis inhibits directed spreading throughout. Inset: Spreading speedup as a function of spreading duration until, like for undirected spreading, almost all bees are “infected”, showing that all three spreading speedups remain negative for longer spreading durations (corresponding prevalence curves in Supplementary Fig. 4a).

To verify that our trophallaxis detector generates plausible results, we used it to monitor three honey bee colonies, each consisting of ~ 1,000 barcoded individuals, at 1 frame/s for five consecutive days. We then employed a temporally explicit epidemiological model to perform *undirected* spreading simulations on the bees’ trophallaxis networks (Table 2). Because these simulations ignore the direction of liquid transfers, they can be used to study information and disease transmission via physical contacts that take place during trophallaxis, such as antennation or touch, but not how the exchanged liquid flows through the colony. Consistent with previous results^8^, we found that undirected spreading through the observed networks of physical contacts was faster than through temporally randomized counterparts (Fig. 3a, Supplementary Figs. 2a and 3a) until most individuals were “infected”, confirming that the temporal structure of these contacts can accelerate the transmission of information or disease^8^.

**Table 2:** Trophallaxis network properties.

Trial	V	E	I	Undirected			Directed		
				*s*_*min*_	*s*_*max*_	*p*_*s*=*0*_	*s*_*min*_	*s*_*max*_	*p*_*s*=*0*_
1	1050	115,269	150,859	− 0.00	6.81	0.97	− 0.19	1.50	0.29
2	902	97,591	132,591	− 0.01	4.99	0.95	− 0.23	1.00	0.26
3	815	60,540	84,164	− 0.04	3.40	0.84	− 0.13	1.18	0.36

For each trial we show the number of nodes (V), edges (E), and trophallaxis interactions (I), the smallest spreading speedup (s_min_), the biggest spreading speedup (s_max_), and the mean prevalence when the speedup of spreading is zero (p_s=0_) for the undirected and the directed spreading simulations. In the case of directed spreading, s_min_, s_max_, and p_s=0_ were computed with respect to the temporally and directionally randomized reference networks.

Knowledge of the direction of liquid transfers allowed us for the first time to also perform *directed* spreading simulations, which model the flow of liquid through the colony (Table 2). These simulations showed that liquid flow was much slower than undirected spreading via physical contacts (Fig. 3, Supplementary Figs. 2, 3), most likely because in directed simulations a recipient cannot “infect” a donor. Moreover, relative to temporally and directionally randomized counterparts, spreading through the observed liquid exchange network was accelerated only during the early phase of the spreading process (Fig. 3b, Supplementary Figs. 2b, 3b); once 0.30 ± 0.05 (mean ± standard deviation) individuals were “infected”, liquid flow was inhibited. By randomizing only either the directionality or the temporal structure of trophallaxis we showed that the temporal structure of trophallaxis is the predominant driver of this dynamic, while its directional nature acts to always inhibit the flow of liquid (Fig. 3b, Supplementary Figs. 2b, 3b). This finding sheds light on the forces that shape the dynamics of this important social behavior in honey bee colonies.

To demonstrate that our approach to automatic behavior detection generalizes to other behaviors, we also developed a detector for egg-laying (Fig. 1b), a solitary behavior performed by worker honey bees when the colony has lost its queen and is unable to replace her^27^. Because, unlike for the above case, we included no proxy for identifying bees that are likely to lay an egg in the region proposal procedure for this detector, it is applied to all bees in an image and therefore generates more false positives. We addressed this problem by adding to the detector a second CNN that was trained to identify and filter out false positives generated by the first CNN. This approach increased the detector’s MCC from 0.64 to 0.76 (see Table 1 for additional performance values). These results demonstrate that our method can be applied to automatically detect very different behaviors.

## Conclusion

We demonstrated that barcode-and-CNN-based detectors accurately identify specific behaviors even under the challenging condition of a dense and populous society. We achieved this by combining information obtained from an individual’s barcode with domain knowledge about the behavior of interest, and showed that our approach can be used to detect very different behaviors.

Behavioral data obtained with one of our new detectors revealed that trophallaxis results in two distinct social networks, each with different transmission dynamics. One network represents physical contacts during trophallaxis and the other network represents liquid transfers among bees. Trophallaxis is a complex behavior that involves multiple sub-behaviors, such as antennation and liquid transfer, and each of these behaviors could serve as a separate transmission channel. Moreover, trophallaxis is known to be involved in different biological processes, such as social learning^28^, the regulation of division of labor^29^, and disease transmission^30^. It is thus not surprising that it results in multiple social networks. However, our finding that the two trophallaxis-related networks exhibit different transmission dynamics was unexpected, and sheds light on how trophallaxis might mediate multiple biological processes simultaneously.

Our approach to automatically detecting behavior can be applied to all animals that perform visually recognizable behaviors, and whose position can be tracked. We therefore envision that this method will make it possible to perform quantitative analysis of behavior in a variety of species, including those that live in large collectives, such as ants, bees, and birds. This will enable automatic, high-resolution behavioral studies that address a broad range of previously intractable questions in evolutionary biology, ethology, neuroscience, and molecular biology.

## Methods

### Honey bee tracking

Colonies were established as in ref.^8^. Briefly, up to 1,370 one-day-old adult honey bee workers (*Apis mellifera*) were individually outfitted with a barcode by chilling them until they stopped moving and then gluing a bCode barcode to their thorax. For recording trophallaxis interactions, we also barcoded an unrelated, naturally mated queen.

All barcoded bees passing quality control checks of barcode placement and readability were moved into a glass-walled observation hive, which was placed in a dark, climate-controlled room and connected to the outside environment via a plastic tube to enable normal foraging. The observation hive held a 348 mm × 232 mm white plastic honeycomb, one side of which was inaccessible to the bees. The other side was provisioned with enough honey and pollen for the duration of the experiment and was covered by an exchangeable glass window. The distance between this window and the honeycomb was short enough to ensure that the bees formed a monolayer, which does not affect their behavior^8^ and prevented them from obscuring their barcodes.

Trophallaxis interactions and egg-laying events were monitored with the tracking systems described in ref.^30^ (Supplementary Fig. 5) and ref.^8^, respectively. Video of the honeycomb was captured for up to 7 consecutive days at a frequency of 1 frame/s with a computer-controlled 29 megapixel monochrome machine vision camera under infrared light, invisible to the bees, and stored on hard drives for later processing. Barcode detection and decoding was performed on a compute cluster, using the software and procedures described in ref.^8^.

### Ground truth

To create a ground truth for the trophallaxis detector, we manually annotated images of pairs of bees that were in the proper position to perform this behavior. For each image we recorded whether the two bees were engaged in trophallaxis and, if so, which bee was the recipient.

The first set of images consisted of the image library *L1* described in ref.^8^. These images show random bee pairs that were selected solely based on whether they were within reach. To determine if two bees are within reach, the coordinate of their mouthparts was estimated by translating the barcode center of each bee by a fixed distance in the direction of her barcode orientation vector, which was assumed to be parallel to her anteroposterior axis (Supplementary Fig. 1). If the distance between these coordinates was shorter than 7 mm, which is the maximum proboscis length of a honey bee^31^, the image was annotated. An image was considered to show trophallaxis if the proboscis of one bee touched the head of the other bee close to her mouthparts (Fig. 2b). Otherwise, it was annotated as not showing trophallaxis.

Examination of the images in library *L1* revealed that only approximately 1 in 40 images showed trophallaxis. Because CNNs learn to classify images better if the number of positive and negative examples is more balanced^32^, and manual annotation is labor intensive, additional images were selected by also requiring that bees needed to face each other, which is necessary for trophallaxis. Whether two bees faced each other was established similar to ref. ^8^: We calculated the angle between the barcode orientation vector of each bee and a line through the mouthpart location of both bees (Supplementary Fig. 1). If the sum of the two resulting angles was less than 104 degrees, the image was annotated. This criterion captures 95% of the trophallaxis contacts in *L1*. Using this filter as a proxy for identifying trophallaxis, we annotated an additional 6045 random bee pair images.

To evaluate the performance of the trophallaxis detector when its predictions are integrated over time, we furthermore used the trophallaxis proxy to annotate all bee pairs in 600 random triples of successively recorded images of the entire observation hive. Each bee pair was annotated by three annotators to be able to reduce annotation errors. Consensus among the three annotators was reached through majority voting. A subset of the resulting annotations was previously published as image library *L2*^8^.

The ground truth for the egg-laying detector was created by manually annotating 1323 random images of the entire hive. Bees that either had inserted their abdomen into a honeycomb cell (Supplementary Fig. 6) or appeared to be in the process of doing so were called egg-layers. All other bees were annotated as having laid no egg. In addition, we annotated each egg layer in up to two images that were recorded before and after these hive images.

The final trophallaxis and egg-laying ground truths consisted of 142,182 images of bee pairs and 723,995 images of individual bees, respectively. Both ground truths were split into disjunct training, calibration, and test data sets as shown in Supplementary Table 4.

### Region proposal

To identify image regions that are likely to show bees engaged in trophallaxis, we leveraged the trophallaxis proxy described earlier (Supplementary Fig. 1). For each pair of potential trophallaxis partners identified by this proxy, we extracted the region showing the heads of both bees by translating the barcode center of each bee by a fixed distance in the direction of her barcode orientation vector. The midpoint of the line segment defined by these two coordinates was used as the center point of a 96 px × 160 px region of which the longer sides were parallel to the aforementioned line segment (Fig. 2b). The top edge of this rectangle was defined to be the short edge closest to the head of the bee with the bigger ID.

For detecting egg-layers, the image region focusing on the abdomen of a bee was extracted by translating the center of a bee’s barcode by a fixed distance in the opposite direction of her barcode orientation vector. The resulting coordinate was used as the midpoint of one edge of a 130 px × 130 px region of which the top edge was parallel and closest to the lower edge of the bee’s barcode (Supplementary Fig. 6). The image region showing the entire bee as well as her immediate surroundings was obtained by first translating the center of her barcode by a fixed distance in the direction of the barcode orientation vector. The resulting coordinate was then used as the top-right corner of a 256 px × 256 px region of which the diagonal between the top-right corner and the bottom-left corner passed through the barcode center (Supplementary Fig. 6).

### Image preprocessing

CNN input images were created by extracting the proposed image region and rotating it “upright”, so its top edge was on the x-axis of the image coordinate system. Pixel intensities were then clamped at a value of 200 to remove bright details in the background that we did not expect to provide information about the behavior of interest, such as the honeycomb structure and specular reflections in the honeycomb cell contents (Fig. 2c). For trophallaxis detection, we furthermore filled the bounding box of the focal bees’ barcode with a uniform color to prevent the CNN from associating parts of the barcode pattern with a behavior (Fig. 2c). Finally, pixel intensities were mean-centered and scaled to [-1, 1] to provide a consistent input range for the CNNs.

### CNN architecture and training

Images of potential trophallaxis partners and egg-layers were classified with two CNNs each. While the exact details of these CNNs varied, they had a similar architecture, consisting of 2 or 3 convolutional layers, max-pooling layers, and 2 fully connected layers (Supplementary Tables 1, 2, and 3). The output of convolutional layers was standardized with batch normalization before being passed to a rectified linear unit activation function, and the output of the final activation function was transformed by a softmax function, so it can be interpreted as the probability of the input image showing the behavior of interest.

CNN training consisted of initializing the weights of the network to values drawn from a normal distribution with a mean of 0 and a standard deviation of 1, truncated at 2 standard deviations. Network weights were then optimized for 10,000 iterations, using the Adam algorithm, which was configured as recommended by its authors (alpha = 0.001, beta1 = 0.9, beta2 = 0.999, epsilon = 0.0000001)^33^, with a cross-entropy loss function. We used batches of 256 images, which were augmented as shown in Supplementary Table 5 and normalized to mean zero and unit variance. To avoid overfitting, we applied a L2 weight decay of 0.005 and a dropout of 0.5 in the first fully connected layer.

The CNNs for detecting the occurrence of trophallaxis and whether a bees’ abdomen was inserted into a honeycomb cell were trained on all positive examples and a matching number of random negative examples from the trophallaxis and egg-laying training data set, respectively. For training the CNN that identifies the recipient, we used all positive examples of the trophallaxis training data set and a corresponding number of random negative examples. The CNN that uses an image of the entire bee to identify false positives was trained on the 4044 false positives generated by the CNN that checks for the visual absence of a bee’s abdomen and a matching number of random positive examples from the egg-laying training data set.

### Behavior detection

To detect trophallaxis or egg-laying in individual images, we first performed the corresponding region proposal to obtain image regions that potentially show the behavior of interest. Each of these regions was extracted, preprocessed as described above, and independently scored by the two CNNs that had been trained to detect the behavior. This approach is computationally less efficient than a strictly serial operation where, for example, the CNN for identifying the recipient processes the input image only if the score for the occurrence of trophallaxis exceeds a specified threshold. It was nevertheless chosen because it makes it possible to store both CNN scores in a file, which can later be post-processed to yield behavior predictions of varying stringency without having to process the video again.

Successive per-image trophallaxis and egg-laying detections were thresholded and linked together to yield behavior predictions that are easier to analyze. Linked predictions were filtered to improve their quality. For trophallaxis, this procedure is identical to the post-processing steps described in ref.^8^. Briefly, detections between the same two individuals were concatenated if they occurred in successive video frames. Concatenated detections shorter than 3 s were discarded, because such short trophallaxis interactions may not always result in the transfer of liquid^34^. The remaining interactions were merged if they were less than 60 s apart, and discarded if their total duration exceeded 180 s. The former threshold is based on the personal observation that trophallaxis partners typically do not engage in trophallaxis again during this time period, while the latter threshold was chosen to be slightly longer than the longest trophallaxis interaction reported in ref.^35^.

For egg-laying, linking and filtering consisted of concatenating the thresholded detections from successive video frames into egg-laying events. Concatenated egg-laying events shorter than 3 s were discarded. The remaining events were merged if they involved the same bee, occurred within 10 s of each other, and the distance of the average position of the events was shorter than 11.2 mm (the width of two honeycomb cells). These conditions ensured that egg-laying predictions were only merged if they appeared to belong to the same real event.

### Detector calibration

The trophallaxis detector and egg-laying detectors each have free parameters. For the trophallaxis detector, these parameters are the minimum and maximum distance between a pair of bees, the maximum sum of the angle between the bees’ orientation vector and a line through their estimated mouthparts location, a threshold for the output of the CNN that determines which scores correspond to the occurrence of trophallaxis, and a threshold for the output of the CNN that identifies the recipient. For the egg-laying detector, free parameters are the CNN thresholds that determine which output scores identify a (potential) egg layer.

To fix the free parameters of each behavior detector, we applied it to the calibration data set of its ground truth. We then performed a grid search on the parameter space of the detector, and chose the parameter combination that maximized the product of the detector’s sensitivity and positive predictive value.

### Detector evaluation

We estimated the fraction of an image that the trophallaxis detector’s region proposal procedure excludes from automatic visual inspection by applying it to the “center” image of all 300 observation hive image triples from which the detector’s test data set was created. We then added the number of pixels inside the proposed image regions and divided the total by the number of pixels per image and by the number of observation hive images.

To test if the CNNs for detecting the occurrence of trophallaxis and for identifying the recipient are sensitive to salient features of the behavior, we performed an occlusion sensitivity analysis^36^. We systematically moved a 41 px × 41 px big grey square (occluder) over the CNN input image, restricting the occluder center to pixels inside the image. For each occluder position, we processed the altered input image with the respective CNN and recorded the CNN’s output. CNN outputs were then spatially coarse-grained into 12 × 20 square bins by averaging over all outputs inside a bin. Bins with a low mean score represent occluder positions that lead to a misclassification of the altered input image.

Detector performance was evaluated by applying each detector to its respective test data set, using the parameters we had obtained during detector calibration. This results in an estimate of a detector’s performance on images of the entire hive, since the prevalence of trophallaxis and egg-laying in the respective test data sets is the same as in hive images. These performance estimates are conservative, because the test data sets consist of 3 s long segments of behavior occurrences that likely lasted longer. Longer behavior occurrences consist of multiple such segments and are therefore more likely to be detected. Moreover, due to the high positive predictive value of both detectors on individual images, the probability of a spurious detection decreases sharply as the duration of the detected behavior increases.

Image processing time was measured by averaging detector runtime across the 900 (545) hive images from which the trophallaxis (egg-laying) test data set was created. These measurements were performed on a cluster with 2.9 GHz Intel Core i9-7920X CPUs and a RAID 6 storage array consisting of 6 HGST H3IKNAS800012872SWW hard drives. During runtime measurements, both detectors were restricted to a single hardware thread and had access to 2 GB RAM.

### Trophallaxis networks

We constructed one temporal network from the trophallaxis detections of each colony. Nodes in these networks represent one bee. Pairs of distinct nodes were connected with a directed edge if the corresponding bees exchanged liquid at least once. A list of elapsed times, counting from the beginning of the experiment, was assigned to each edge to specify when the liquid transfers were initiated. These contact initiation times had a resolution of 1 s and enabled our spreading simulations to maintain the precise time-order of transfer events.

### Spreading simulations

To simulate the transmission of information, pathogens, and liquid, we employed a temporally explicit version of the deterministic susceptible-infected model^37^. This model assumes that individuals are in one of two states, “susceptible” or “infected.” Simulations begin by setting all bees to susceptible, choosing a trophallaxis interaction uniformly at random, and “infecting” the two bees involved in this interaction, independent of how long they had interacted. Spreading dynamics were then simulated over an 8 h time window. During this time, an infected donor infects a susceptible recipient with a probability of 1 when they engage in trophallaxis (directed transmission). For models of undirected transmission, the trophallactic role of individuals was ignored, which means recipients were also able to infect donors.

For each simulation, we recorded the fraction of infected individuals *f*(*t*) = *i*(*t*)/*S*(*t*), where *i*(*t*) is the number of infected bees alive at time *t* after the first infection, and *S*(*t*) is the colony size at time *t*. To obtain a more robust estimate of the fraction of infected individuals, we averaged it over *R* = 1000 simulation runs and calculated the prevalence $p\left(t\right)=\frac{{\sum }_{r=1}^{R}{f}_{r}\left(t\right)}{R}.$

To establish whether the prevalence for an observed interaction sequence, *p̂*(*t*), is greater than expected by chance, we compared it to the prevalence for *N* = 5 randomized interaction sequences. For undirected spreading simulations, randomized interaction sequences were created with the PTN null model^38^, which shuffles the contact times among the observed interactions while ensuring that no individual gets assigned an interaction after its time of death. For directed spreading simulations, interaction sequences were randomized by reversing the direction of trophallaxis with a probability of 0.5 before the PTN null model was applied. To separately study the effect of the directional nature and of the temporal structure of trophallaxis on directed spreading, we performed only one of these randomizations, respectively. Spreading dynamics where characterized by calculating the spreading speedup $s\left( t \right)\, = \,\left( {\hat{p}\left( t \right) - \overline{p}\left( t \right)} \right)/{\text{min}}\left( {\hat{p}\left( t \right),\overline{p}\left( t \right)} \right)$, where $\overline{p }\left(t\right)={\sum }_{n=1}^{N}\frac{{p}_{n}\left(t\right)}{N},$ is the mean prevalence across the *N* randomized interaction sequences.

## Supplementary Information

Supplementary Information.

## Data Availability

The datasets generated and analyzed during the current study are available in Zenodo, https://doi.org/10.5281/zenodo.5582779.

## Code availability

Computer code for detecting trophallaxis and egg-laying is available in Zenodo, https://doi.org/10.5281/zenodo.5584131.
