# Supplementary material for: Automated monitoring of honey bees with barcodes and artificial intelligence reveals two distinct social networks from a single affiliative behavior
Source: Sci Rep. 2023 Jan 27;13:1541. doi: 10.1038/s41598-022-26825-4 (PMC9883485; doi:10.1038/s41598-022-26825-4)
Supplement: Supplementary file 1 — Supplementary Information. [file 41598_2022_26825_MOESM1_ESM.docx]

*Supplementary Information for*

**Automated monitoring of honey bees with barcodes and artificial intelligence reveals two distinct social networks from a single affiliative behavior**

Tim Gernat^a,b^, Tobias Jagla^b^, Beryl M. Jones^a,†^, Martin Middendorf^b^, Gene E. Robinson^a,c,d,*^

^a^Carl R. Woese Institute for Genomic Biology, University of Illinois at Urbana-Champaign, 1206 West Gregory Drive, Urbana, IL 61801, USA; ^b^Swarm Intelligence and Complex Systems Group, Department of Computer Science, Leipzig University, Augustusplatz 10, 04109 Leipzig, Germany; ^c^Neuroscience Program, University of Illinois at Urbana-Champaign, 505 South Goodwin Avenue, Urbana, IL 61801, USA; ^d^Department of Entomology, University of Illinois at Urbana-Champaign, 320 Morrill Hall, Urbana, IL 61801, USA

^†^Present affiliations: Department of Ecology and Evolutionary Biology, Princeton University, 106A Guyot Lane, Princeton, NJ 08544, USA; Lewis-Sigler Institute for Integrative Genomics, Princeton University, South Drive, Princeton, NJ 08544, USA

*Corresponding author: Gene E. Robinson, Carl R. Woese Institute for Genomic Biology, University of Illinois at Urbana-Champaign, 1206 West Gregory Drive, Urbana, IL 61801, USA; +1-217-2650309; generobi@illinois.edu

# Supplementary Figures


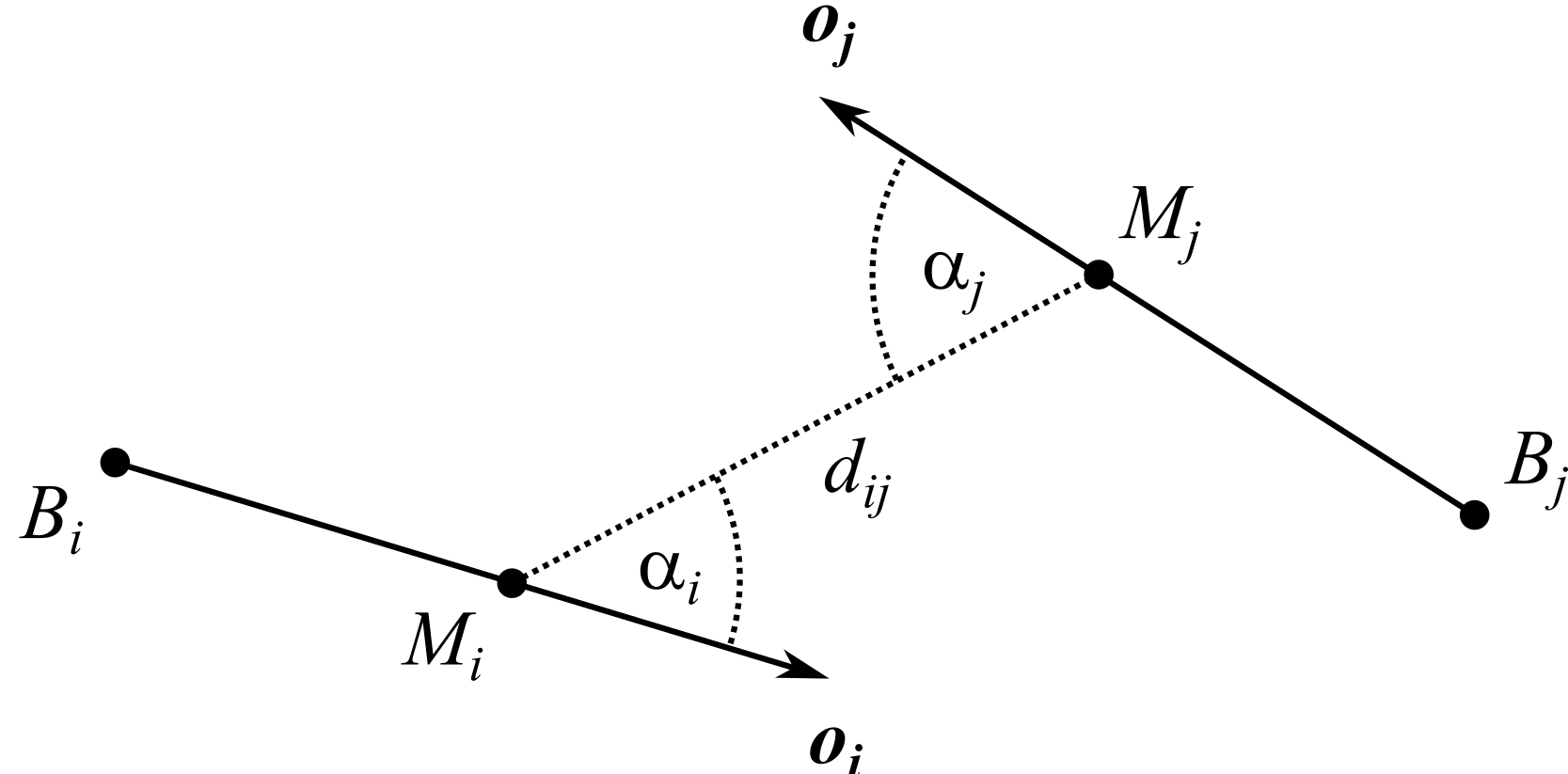


Supplementary Figure 1 | Spatial proxy for identifying pairs of bees that might be engaged in trophallaxis. Points *B_i_* and *B_j_* are the barcode centers of bee *i* and *j*, respectively. Arrows represent the barcode orientation vectors *o_i_* and *o_j_* that correspond to the heading direction of a bee. Points *M_i_* and *M_j_* are the estimated mouthparts locations of the two bees, and *d_ij_* is the distance between these points. If *d_ij_* was shorter than the proboscis length of a honey bee and the sum of the angles α*_i_* and α*_j_* is smaller than a specified threshold, bees *i* and *j* were called potential trophallaxis partners.


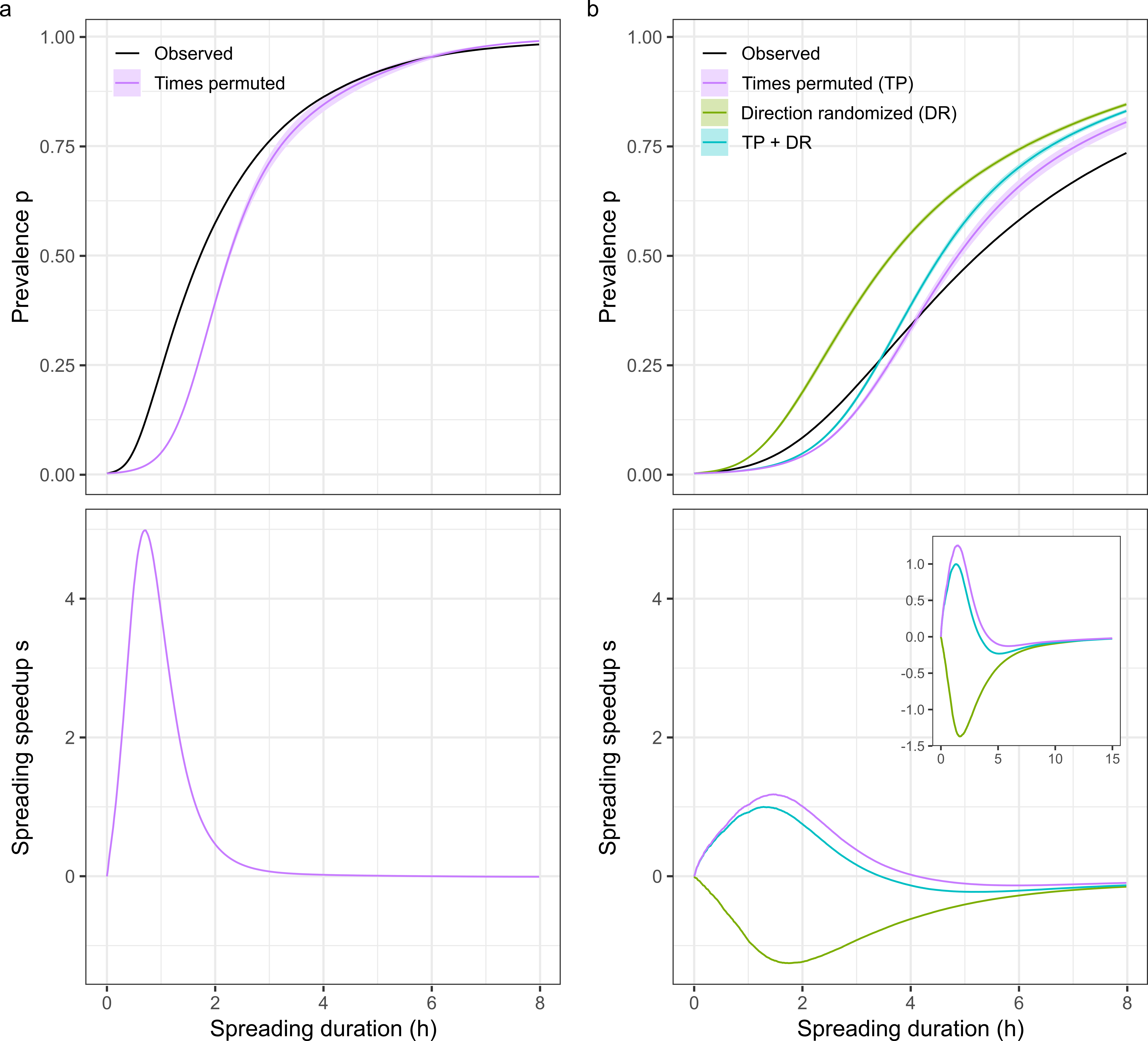


Supplementary Figure 2 | Simulated spreading in honey bee trophallaxis networks. Panels show data from Trial 2. a, Undirected spreading results, modeling transmission via physical contacts during trophallaxis. Top panel: Prevalence as a function of spreading duration. Black line, prevalence in the observed trophallaxis network; magenta line, mean prevalence, averaged across 5 temporally randomized reference networks; magenta band, point-wise 95% confidence interval. Bottom panel: Spreading speedup as a function of spreading duration. b, Directed spreading results, modeling liquid flow. Panels as in a. Green (turquoise), prevalence and spreading speedup in directionally (temporally and directionally) randomized reference networks. Inset: Spreading speedup as a function of spreading duration until almost all bees are “infected” (corresponding prevalence curves in Supplementary Fig. 4b).


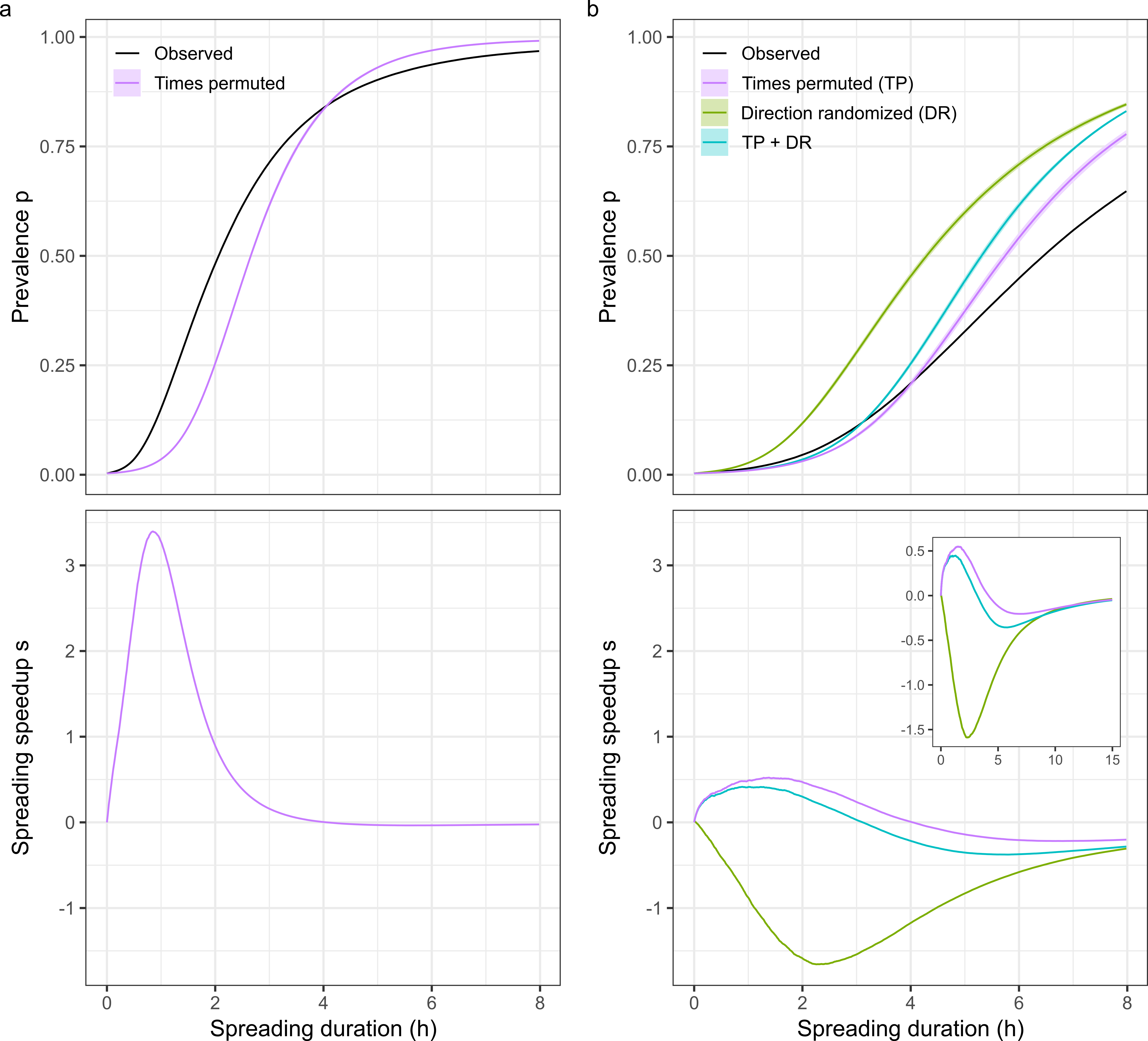


Supplementary Figure 3 | Simulated spreading in honey bee trophallaxis networks. Panels show data from Trial 3. a, Undirected spreading results, modeling transmission via physical contacts during trophallaxis. Top panel: Prevalence as a function of spreading duration. Black line, prevalence in the observed trophallaxis network; magenta line, mean prevalence, averaged across 5 temporally randomized reference networks; magenta band, point-wise 95% confidence interval. Bottom panel: Spreading speedup as a function of spreading duration. b, Directed spreading results, modeling liquid flow. Panels as in a. Green (turquoise), prevalence and spreading speedup in directionally (temporally and directionally) randomized reference networks. Inset: Spreading speedup as a function of spreading duration until almost all bees are “infected” (corresponding prevalence curves in Supplementary Fig. 4c).


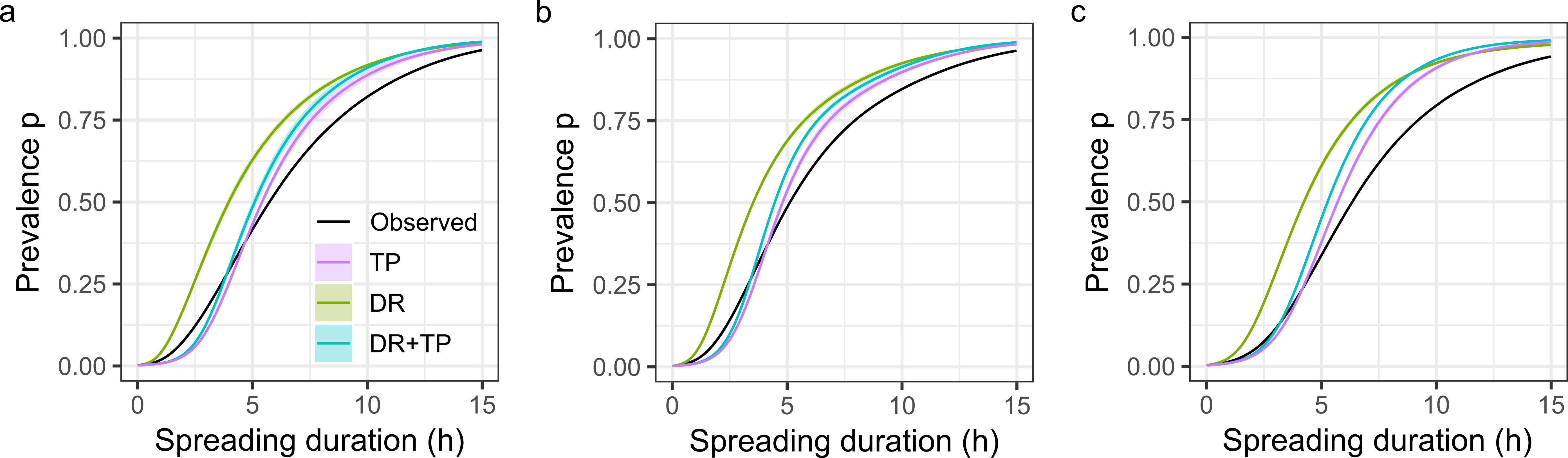


Supplementary Figure 4 | Prevalence until almost all bees are “infected” during directed spreading simulations. Black line, prevalence in the observed trophallaxis network; magenta, green, and turquoise lines, mean prevalence, averaged across 5 temporally (TP), directionally (DR), and temporally and directionally (TP+DR) randomized reference networks, respectively; bands are point-wise 95% confidence intervals. a, Trial 1. b, Trial 2. c, Trial 3.


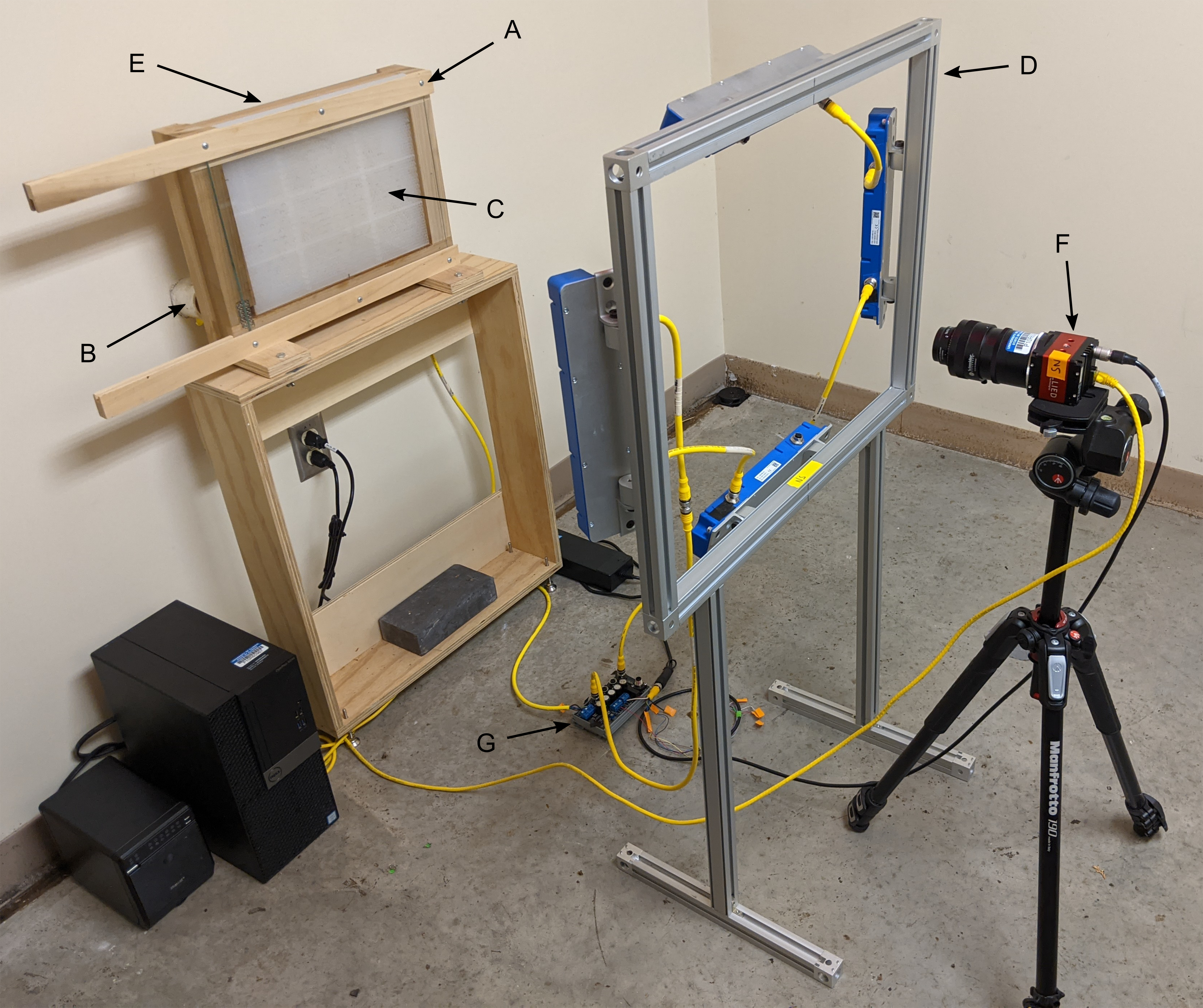


Supplementary Figure 5 | Setup for tracking barcoded honey bees and automatically monitoring their behavior. Barcoded bees were housed in an observation hive (A) that was connected to the outdoors via an entrance tube (B). The hive held a glass-covered, one-sided plastic honeycomb (C), which was front-lit with four infrared LED lights mounted on an aluminum frame (D) and backlit with an array of infrared lights mounted behind the hive (E, hidden). A computer-controlled high-resolution monochrome camera (F) recorded the hive, triggering the infrared lights via a breakout board (G).


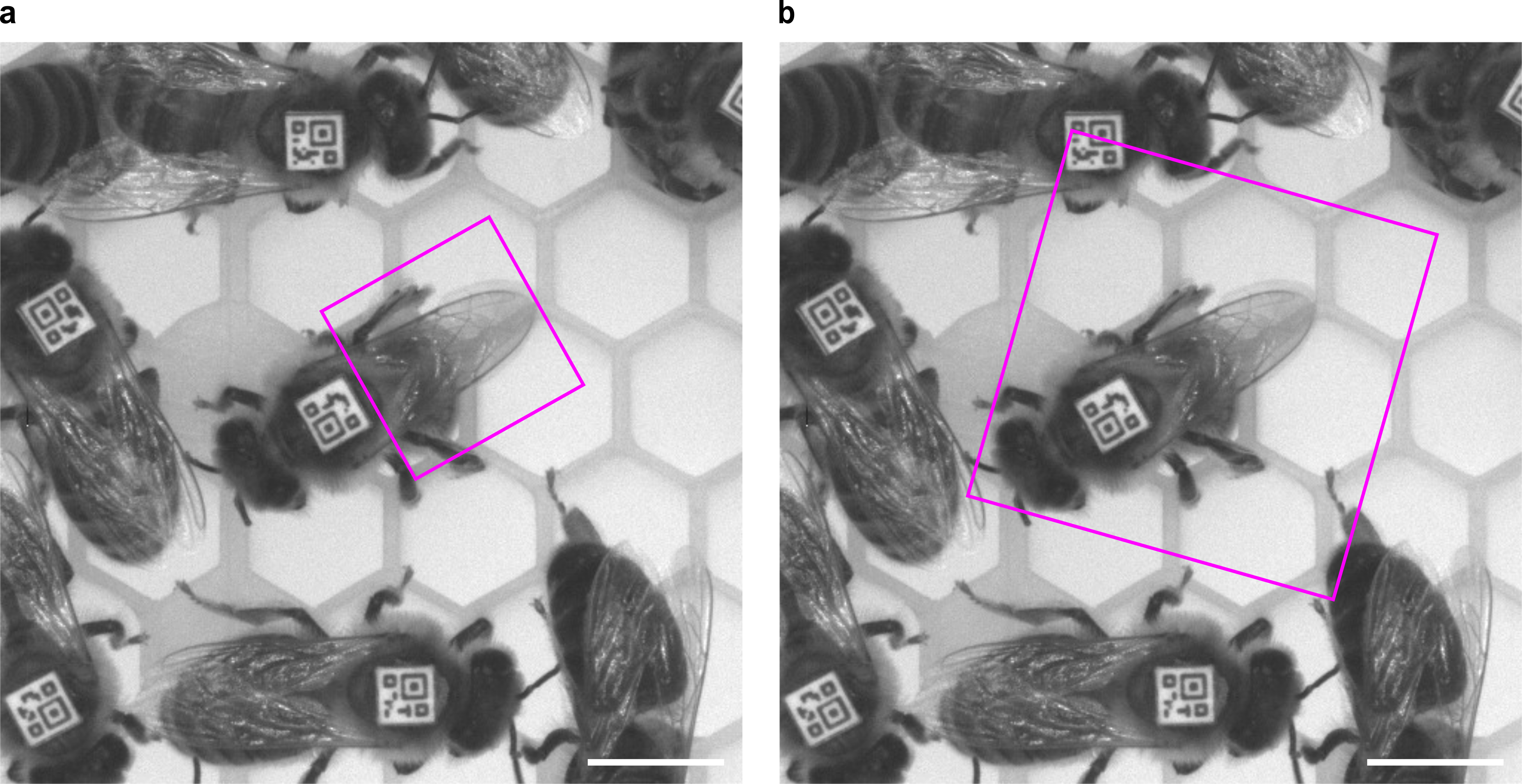


Supplementary Figure 6 | Region proposals for the egg-laying detector. Proposed image regions are shown as magenta rectangles. a, Image region focusing on the bee’s abdomen, which is invisible because the bee has inserted it into a honeycomb cell to position an egg. This region was used to identify potential egg-layers. Scale bar, 5 mm. b, Image region of the entire bee. This region was used to classify potential egg-layers into true egg-layers and false positives. Scale bar, 5 mm.

# Supplementary Tables

| **Layer** | **Layer type** | **Kernel size** | **Stride** | **Output dimensions** |
| --- | --- | --- | --- | --- |
| 0 | I | - | - | 36 × 60 × 1 |
| 1 | C | 5 × 5 | 1 | 36 × 60 × 8 |
| 2 | MP | 2 × 2 | 2 | 18 × 30 × 8 |
| 3 | C | 3 × 3 | 1 | 18 × 30 × 16 |
| 4 | MP | 2 × 2 | 2 | 9 × 15 × 16 |
| 5 | F | - | - | 1 × 1 × 32 |
| 6 | F | - | - | 1 × 1 × 2 |

Supplementary Table 1 | Architecture of the CNN for predicting the occurrence of trophallaxis and of the CNN for identifying the recipient. I: input layer; C: convolutional layer; MP: max-pooling layer; F: fully connected layer. Layer output dimensions are W × H × D.

| **Layer** | **Layer type** | **Kernel size** | **Stride** | **Output dimensions** |
| --- | --- | --- | --- | --- |
| 0 | I | - | - | 32 × 32 × 1 |
| 1 | C | 3 × 3 | 1 | 32 × 32 × 32 |
| 2 | MP | 2 × 2 | 2 | 16 × 16 × 32 |
| 3 | C | 5 × 5 | 1 | 16 × 16 × 64 |
| 4 | MP | 2 × 2 | 2 | 8 × 8 × 64 |
| 5 | F | - | - | 1 × 1 × 256 |
| 6 | F | - | - | 1 × 1 × 2 |

Supplementary Table 2 | Architecture of the CNN for predicting whether a bee has inserted her abdomen into a honeycomb cell. I: input layer; C: convolutional layer; MP: max-pooling layer; F: fully connected layer. Layer output dimensions are W × H × D.

| **Layer** | **Layer type** | **Kernel size** | **Stride** | **Output dimensions** |
| --- | --- | --- | --- | --- |
| 0 | I | - | - | 64 × 64 × 1 |
| 1 | C | 5 × 5 | 1 | 64 × 64 × 32 |
| 2 | MP | 2 × 2 | 2 | 32 × 32 × 32 |
| 3 | C | 3 × 3 | 1 | 32 × 32 × 64 |
| 4 | MP | 2 × 2 | 2 | 16 × 16 × 64 |
| 5 | C | 3 × 3 | 1 | 16 × 16 × 128 |
| 6 | MP | 2 × 2 | 2 | 8 × 8 × 128 |
| 7 | F | - | - | 1 × 1 × 256 |
| 8 | F | - | - | 1 × 1 × 2 |

Supplementary Table 3 | Architecture of the CNN for classifying the predictions generated by the CNN shown in Supplementary Table 2 into true egg-layers and false positives. I: input layer; C: convolutional layer; MP: max-pooling layer; F: fully connected layer. Layer output dimensions are W × H × D.

| **Behavior** | **Image subset** | **Annotators per image** | **Successive time points** | **Positive examples** | **Negative examples** |
| --- | --- | --- | --- | --- | --- |
| Trophallaxis | Training | 1 | 1 | 2090 | 43828 |
|  | Calibration | 3 | 3 | 993 | 38832 |
|  | Test | 3 | 3 | 1581 | 54858 |
| Egg-laying | Training | 1 | 5 | 5539 | 319078 |
|  | Calibration | 1 | 3 | 639 | 201888 |
|  | Test | 1 | 3 | 729 | 196122 |

Supplementary Table 4 | Overview of the trophallaxis and egg-laying gold standards. The number of successive time points indicates if image sequences were annotated, and how long these sequences were. The number of positive and negative examples shows the number of images per subset. The number of annotations per subset is therefore higher than the number of images per subset if multiple annotators scored each image.

| **Technique** | **Probability** | **Trophallaxis** | | **Egg-laying** | |
| --- | --- | --- | --- | --- | --- |
|  |  | Trophallaxis detection | Recipient identification | Abdomen detection | Pose examination |
| Adjust brightness | 1 | + | + | + | + |
| Adjust contrast | 1 | + | + | + | + |
| Jitter | 1 | + | + | + | + |
| Flip vertical | 0.5 | + | + | + | - |
| Flip horizontal | 0.5 | + | + | - | - |
| Flip diagonal | 0.5 | - | - | - | + |

Supplementary Table 5 | Overview of the data augmentation techniques used to increase the diversity of the data sets for training the two CNNs employed by the trophallaxis detector and the egg-laying detector. A technique was applied to each example image with the specified probability if the corresponding table cell shows a plus sign; otherwise, the technique was not used.
